# The structure of rhizosphere microbial and endophytic communities of *Coptis chinensis* var. *brevisepala*: variations across different ecological niches

**DOI:** 10.3389/fmicb.2026.1785609

**Published:** 2026-03-18

**Authors:** Cuiting Chen, Pan Wang, Genping Tong, Rubing Chen, Yanghui Shen, Xiaojun Wu, Weiqing Liang, Jinbao Pu

**Affiliations:** 1 Center for Medicinal Resources Research, Zhejiang Academy of Traditional Chinese Medicine, Hangzhou, Zhejiang, China; 2 Zhejiang Key Discipline in Traditional Chinese Medicine for Pharmaceutical Botany, Hangzhou, Zhejiang, China; 3 Zhejiang Engineering Research Center for Quality Assessment and Development of Dao-di Herbs, Hangzhou, Zhejiang, China; 4 Pan’an Traditional Chinese Medicine Industry Innovation and Development Institute, Jinhua, China; 5 Qingliangfeng National Nature Reserve of Zhejiang, Linan, Zhejiang, China

**Keywords:** community composition, *Coptis chinensis* var. *brevisepala*, endophytes, rhizosphere microorganisms, soil factors

## Abstract

*Coptis chinensis* var. *brevisepala* is a valuable traditional Chinese medicinal plant, whose resources are severely depleted due to long-term overexploitation. However, the associations between its rhizosphere microbiome and habitat soil properties, as well as the composition and functions of endophytes, remain unclear. This study employed high-throughput sequencing to characterize rhizosphere microbial communities of *C. chinensis* var. *brevisepala* from four distribution sites, analyze their correlations with soil chemical properties, and explore the differences and functional traits of endophytic communities in distinct tissues (leaves, rhizomes, fibrous roots). A total of 177 core bacterial genera and 146 core fungal genera were detected in rhizosphere soils of the four sites. The dominant bacterial phyla were Proteobacteria, Acidobacteriota, and Actinobacteriota, with norank_f_Xanthobacteraceae and *Bradyrhizobium* as the dominant genera. The dominant fungal phyla were Ascomycota and Basidiomycota, with *Paraboeremia* and *Saitozyma* as the dominant genera. Soil chemical properties exerted significant effects on both bacterial and fungal communities in the rhizosphere, among which soil pH and total nitrogen (TN) were the key drivers shaping rhizosphere microbial communities. For endophytes, 29 bacterial phyla (596 genera) and 12 fungal phyla (653 genera) were identified, with significant differences in diversity, richness, and dominant genera across tissues; leaves harbored the highest endophytic diversity. Functional prediction indicated that endophytic fungi were dominated by saprotrophy-related functional genes, and KEGG secondary functional annotation uncovered the presence of antimicrobial-related genes. This study clarifies the rhizosphere microbiome ecological traits and tissue-specific endophytic characteristics of *C. chinensis* var. *brevisepala*, providing a scientific basis for screening beneficial microorganisms to facilitate the restoration and reconstruction of this endangered medicinal plant.

## 1 Introduction

*Coptis chinensis* var. *brevisepala* (*C. chinensis* var. *brevisepala*), historically referred to as “Xuanhuanglian”—a renowned traditional Chinese medicinal herb, has long been highly valued by practitioners due to its exceptional pharmaceutical properties (Ao et al., 2021; Zhou et al., 2025). Its rhizomes are rich in alkaloids such as berberine, which exhibit therapeutic effects on bacillary dysentery, tuberculosis, typhoid, epidemic cerebrospinal meningitis, and pertussis (Zhang and Zhang, 2005; Mao and Liu, 2012; Wang et al., 2021). However, due to long-term over-exploitation and habitat destruction, resources of this species have declined sharply, rendering it extremely rare in natural settings with only fragmented, sporadic distributions persisting (Liu et al., 2010). Numerous studies have explored the endangerment mechanisms of *C. chinensis* var. *brevisepala* by investigating its population characteristics, habitat conditions, reproductive strategies, and biological traits. These studies have confirmed that *C. chinensis* var. *brevisepala* functions as an associated species in plant communities, with wild populations characterized by low abundance, small population size, and structural instability (Zhang and Zhang, 2005). Under natural conditions, *C. chinensis* var. *brevisepala* is primarily distributed in valley forests or riparian understories at elevations of 600–1,200 m, with highly specialized habitat requirements—high shading intensity, high humidity, and well-drained soils (Chen and Liu, 2012). And such specialized habitats have also shaped unique microbial environments, which are likely to be closely associated with the survival and adaptation of *C. chinensis* var. *brevisepala*. Therefore, a systematic elucidation of the ecological traits of *C. chinensis* var. *brevisepala*, particularly the associated microorganisms tightly linked to its survival and adaptation, is of great significance for the scientific conservation and sustainable utilization of this species.

Over hundreds of millions of years, plants and their associated microorganisms have coevolved, gradually establishing symbiotic systems with tightly integrated functions. This symbiotic interaction has emerged as a key component in sustaining plant fitness and ecosystem stability through various mechanisms (Rai and Agarkar, 2016; Trivedi et al., 2020). Beneficial microbes, including plant growth promoting rhizobacteria (PGPR) and endophytic fungi, can secrete secondary metabolites (e.g., organic acids, IAA, siderophores, antibiotics) and effectors to directly or indirectly facilitate nutrient acquisition, promote plant growth, and inhibit pathogens (Hiruma et al., 2016; Berendsen et al., 2018; Zhuang et al., 2024). For instance, soil microbes can intercept plant hormones, thereby attenuating the plant stress response to drought (Fitzpatrick et al., 2018). Additionally, microorganisms also have pronounced ecological ramifications. Soil microorganisms are not only involved in nutrient cycling and organic matter transformations but also alter many other soil habitats through various biochemical and biophysical mechanisms (Stursová et al., 2012; Tláskal et al., 2016; Herzog et al., 2019; Philippot et al., 2023). In recent years, with the accumulation of research, inoculating plants with PGPR or utilizing microbe-to-plant signal compounds has become effective strategies to promote plant yield and quality and enhance plant tolerance to more frequent abiotic stresses under intensifying climate change (Backer et al., 2018; Zaheer et al., 2024). Thus, research on plant-associated microbial communities provides support for developing microbe-based green plant protection technologies and exhibits broad application prospects in fields including plant protection.

Plant genotypes and geographical location are well recognized to play pivotal roles in shaping the root-associated microbiota (Stopnisek and Shade, 2021; Sun et al., 2022). For tropical tree species. Studies have demonstrated that the regulatory effect of geographical location on bacterial community composition and diversity even exceeds the inherent genetic traits of the host plants themselves (Kristensen et al., 2021; Ji et al., 2024). Xu H. et al. (2024) characterized the structure and diversity of peach-tree rhizosphere soil microbial communities, and observed variations in bacterial diversity and community composition across three peach-producing areas in Linyi. The underlying mechanism is that distinct geographical locations are typically accompanied by marked differences in soil properties, temperature, and others, which further select for and shape geographically specific microbial communities. Numerous studies have demonstrated that soil properties can influence microbial communities, including altering the diversity and relative abundance of both bacterial and fungal communities (Fierer, 2017; Philippot et al., 2023). However, the soil properties and their functional roles in the microbiota associated with *C. chinensis* var. *brevisepala* are still poorly understood.

As a rare and endangered medicinal plant endemic to China, *C. chinensis* var. *brevisepala*’s survival and habitat maintenance may be highly dependent on interactions with microorganisms. However, our current understanding of the microbiota of *C. chinensis* var. *brevisepala*, as well as soil factors shaping its microbiota, remains limited; yet this knowledge is essential for understanding this species’ survival strategies. This study aims to characterize the structural features of rhizosphere microbial communities of *C. chinensis* var. *brevisepala* across distinct distribution sites and predict the potential functions of these microorganisms; explore how soil chemical properties shape the diversity and composition of its rhizosphere microbial communities; and assess the diversity, community structure, and key microbial taxa of endophytes in different tissues (leaves, rhizomes, and fibrous roots) of *C. chinensis* var. *brevisepala*, and examine the relationships among tissue-specific endophytic communities. The findings will provide essential support for formulating conservation strategies for *C. chinensis* var. *brevisepala* and its habitat ecosystem.

## 2 Materials and methods

### 2.1 Study location and sample collection

The samples for this study were collected in 2024 from 4 distribution sites of *C. chinensis* var. *brevisepala* in Zhejiang Province, China (27°02′–31°11′N, 118°01′–123°10′E). Zhejiang has a subtropical monsoon climate, with an annual average temperature of 15–18 °C and annual precipitation of 1,100–2,000 mm. The 4 sampling sites were strategically selected across different elevations of *C. chinensis* var. *brevisepala* habitats, with their geographical coordinates and elevations as follows: PA (28°58′N, 120°33′E, 681 m), LTS (30°1′N, 118°56′E, 905 m), GHZ (30°7′N, 118°54′E, 1,029 m), and HH (31°8′N, 120°50′E, 726 m). All sites are located in evergreen-deciduous broad-leaved forests, where *C. chinensis* var. *brevisepala* grows as an associated species.

At each site, 5 independent rhizosphere soil samples were collected randomly. Sampling procedures were as follows: plant roots were excavated, and most soil was removed by shaking until no loose soil remained; the soil tightly adhering to root surfaces was gently brushed off using sterile soft brushes. Each soil sample was divided into two parts: one part was flash-frozen in liquid nitrogen, transported to the laboratory on dry ice, and stored at −80 °C for subsequent microbial community analysis; the other part was air-dried naturally for determination of soil physicochemical properties. For plant tissue samples, tissues from 5 *C. chinensis* var. *brevisepala* individuals at the GHZ site were collected. After surface cleaning, sterilization was performed in a sterile ultra-clean bench: samples were washed with sterile water for 30 s, soaked in 75% ethanol for 2 min, 2.5% NaClO for 5 min, and 75% sterile ethanol for 30 s, followed by 3–5 rinses with sterile water. Sterilized samples were stored at −80 °C until use.

### 2.2 DNA extraction and PCR amplification

Total genomic DNA of microbial communities was extracted using the E.Z.N.A.^®^ Soil DNA Kit (Omega Bio-tek, Norcross, GA, USA) following the manufacturer’s instructions. After assessing DNA quality, purity, and concentration, qualified DNA was used as a template for PCR amplification. The 16S rRNA gene V3–V4 region was amplified using universal primers 338F (5′-ACTCCTACGGGAGGCAGCAG-3′) and 806R (5′-GGACTACHVGGGTWTCTAAT-3′); the ITS region was amplified using universal primers ITS1F (5′-CTTGGTCATTTAGAGGAAGTAA-3′) and ITS2R (5′-GCTGCGTTCTTCATCGATGC-3′). The PCR reaction system (20 μL total) contained: 10 μL of 5 × Pro Taq, 0.8 μL of forward primer (5 μmol∙L^−1^), 0.8 μL of reverse primer (5 μmol∙L^−1^), 10 ng of template DNA, and nuclease-free water to make up the volume. Amplification conditions were: initial denaturation at 95 °C for 3 min; 27 cycles of denaturation at 95 °C for 30 s, annealing at 55 °C for 30 s, and extension at 72 °C for 45 s; final extension at 72 °C for 10 min; and holding at 4 °C.

### 2.3 Illumina sequencing and data processing

Libraries of PCR products were constructed using the NEXTFLEX^®^ Rapid DNA-Seq Kit, involving: adapter ligation; removal of adapter-dimer fragments via magnetic bead screening; library template enrichment by PCR; and PCR product recovery using magnetic beads to obtain final libraries. Sequencing was performed on the Illumina Nextseq2000 platform by Majorbio Bio-pharm Technology Co., Ltd. (Shanghai, China). Raw paired-end sequencing data were quality-filtered using Fastp^1^ and merged using FLASH.^2^ Optimized sequences after quality control and merging were denoised using the DADA2 plugin in the QIIME2 (v2024) with default parameters. Denoised sequences were referred to as amplicon sequence variants (ASVs). Sequences annotated as chloroplasts or mitochondria were removed from all samples. To minimize the impact of sequencing depth on subsequent alpha and beta diversity analyses, all samples were rarefied to 20,000 sequences, resulting in an average Good’s coverage of 97.90% per sample. Taxonomic classification of ASVs was performed using the Naive Bayes classifier in QIIME2 against the Silva 16S rRNA gene database (v138).

Functional profiles of the rhizosphere bacterial communities were predicted using PICRUSt2 (v2.2.0) based on 16S rRNA gene amplicon sequencing data. Briefly, the ASV table was first normalized by the 16S rRNA gene copy numbers of corresponding taxa to account for copy number variation across bacterial genomes. The normalized ASV table was then mapped to the Kyoto Encyclopedia of Genes and Genomes (KEGG) database to infer the functional gene content of the bacterial community. The predicted functions were further categorized into KEGG Level 2 and Level 3 pathways for subsequent statistical analysis and functional interpretation. Fungal functional annotation was performed using FUNGuild (v1.0) to assign ecological functions to fungal ASVs based on UNITE-derived taxonomic classifications. ASVs with <93% sequence similarity to reference sequences were excluded prior to annotation. The QIIME-formatted ASV table was mapped to the FUNGuild database, with redundant matches dereplicated by retaining the most finely resolved taxonomic rank. Only annotations with “highly probable” or “probable” confidence were retained, and ASVs were categorized into three trophic modes (pathotrophs, symbiotrophs, saprotrophs) and corresponding ecological guilds to characterize the ecological roles of rhizosphere fungi (Nguyen et al., 2016).

### 2.4 Determination of soil chemical properties

Soil pH was measured at a ratio of 1:2.5 soil/water mixtures. Soil organic matter (SOM) content was determined according to the method of Nelson and Sommers (1982). Soil total nitrogen (TN) was measured by the Kjeldahl method. Soil total phosphorus (TP) and total potassium (TK) were determined by acid solubilization (Miswan et al., 2023). Available nitrogen (AN) was determined according to the method of Ju et al. (2020). Soil available phosphorus (AP) was extracted with sodium bicarbonate and then determined using the molybdenum blue method (Jia et al., 2015). Available potassium (AK) was extracted and analyzed by ammonium acetate and Flame Photometry (Helmke and Sparks, 1996).

### 2.5 Statistical analysis

All data analyses were performed on the Majorbio Cloud Platform.^3^ Alpha diversity indices, including Abundance-based Coverage Estimator (ACE) and Shannon, were calculated using mothur.^4^ Venn diagram were generated using 97% sequence-similarity ASV/taxonomic abundance tables, with shared and unique taxa across samples quantified and visualized in R (v3.3.1). Principal Coordinates Analysis (PCoA) based on Bray-Curtis distances was used to assess similarities in microbial community structure among samples, with permutational multivariate analysis of variance (PERMANOVA) to test the statistical significance of differences between groups. Linear discriminant analysis Effect Size (LEfSe)^5^ with LDA > 4 and *p* < 0.05 was used to identify microbial taxa with significantly different abundances between groups at phylum to genus levels. Redundancy analysis (RDA) was performed to explore the effects of soil physicochemical properties on microbial community structure, and Spearman’s correlation analysis (*p* < 0.05) was used to construct heatmaps of correlations between species and soil properties. Mean values and standard deviations were calculated using SPSS 27 (SPSS, Chicago, USA). One-way analysis of variance (ANOVA) with Tukey’s HSD test was used to assess significant differences between groups, with statistical significance defined as *p* < 0.05. Data visualization was performed using GraphPad Prism 8.0.

## 3 Results

### 3.1 Composition and relative abundance of the rhizosphere core microbial communities

**Figure 1:**
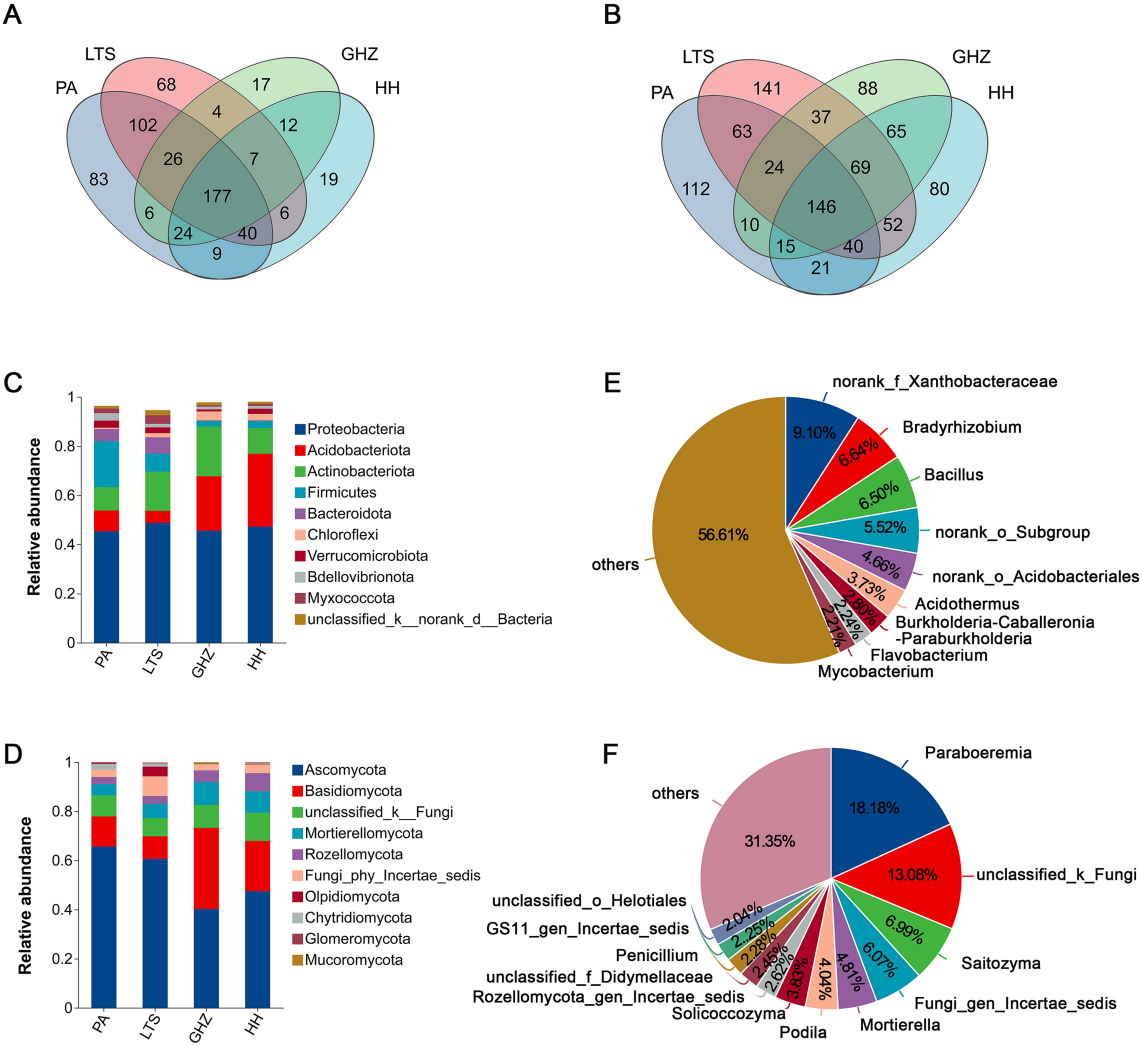
Composition of rhizosphere microbial communities of *C. chinensis* var. *brevisepala* across different distribution sites (PA, LTS, GHZ, HH). **(A)** Venn diagram of bacterial communities at the genus level; **(B)** Venn diagram of fungal communities at the genus level; **(C,D)** The relative abundance of bacteria **(C)** and fungi **(D)** at the phylum level; **(E,F)** The relative abundance of bacteria **(E)** and fungi **(F)** at the genus level (abundance > 2%).

Core rhizosphere microorganisms, exerts a pivotal effect on plant growth, was defined as microbial taxa shared across four distinct distribution sites (PA, LTS, GHZ, HH) and present independent of geographic location. Venn diagrams (Figures 1A,B) showed that the rhizosphere core bacterial microbiota comprised 177 genera (belonging to 20 phyla) and the core fungal microbiota comprised 146 genera (belonging to 11 phyla). The dominant core bacterial phyla comprised Proteobacteria, Acidobacteriota, Actinobacteriota, Firmicutes, and Bacteroidota, while the dominant core fungal phyla included Ascomycota, Basidiomycota, unclassified_k_Fungi, Mortierellomycota, and Rozellomycota (Figures 1C,D). At the genus level, the core bacteria were predominantly norank_f_Xanthobacteraceae (9.10%), *Bradyrhizobium* (6.64%), *Bacillus* (6.50%), norank_o_Subgroup_2 (5.52%), and norank_o_Acidobacteriales (4.66%); the core fungi were mainly *Paraboeremia* (18.18%), unclassified_k_Fungi (13.08%), *Saitozyma* (6.99%), Fungi_gen_Incertae_sedis (6.07%), and *Mortierella* (4.81%) (Figures 1E,F).

### 3.2 Diversity analysis of the rhizosphere microbial communities

**Figure 2:**
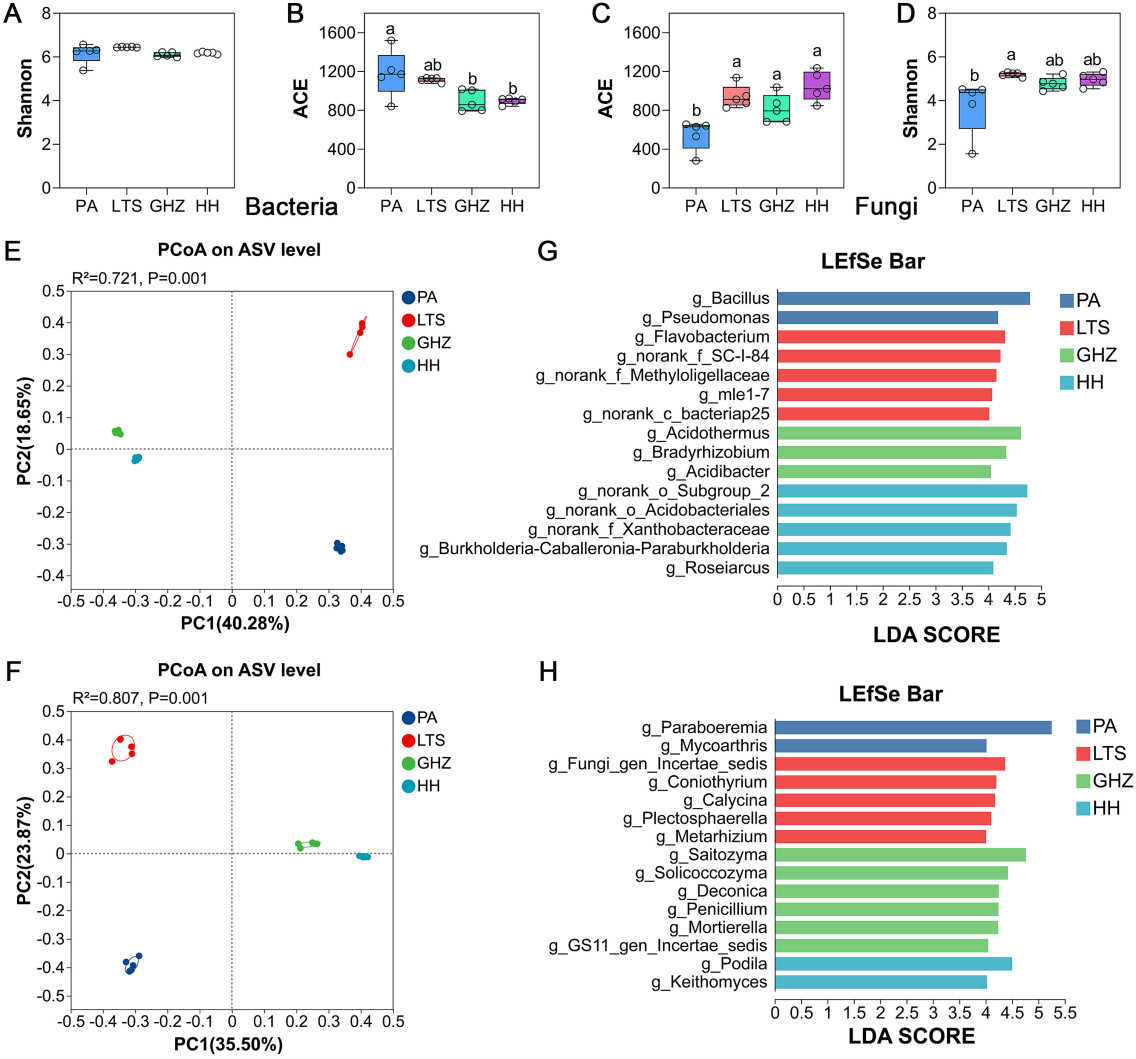
Bacterial and fungal community profiles in the rhizosphere soil of *C. chinensis* var. *brevisepala* across multiple sites. **(A–D)** Alpha diversity index; **(E,F)** PCoA analysis of bacteria **(E)** and fungi **(F)**. **(G,H)** LEfSe analysis of bacteria **(G)** and fungi **(H)** with an LDA threshold of >4. Groups at genus levels were determined to be significant representations of their sample group. An all-against-all comparison in multiclass analysis was performed. Each variable was ranked based on the size of its effects in each sample. When compared among samples, habitat was identified as significant (*p* < 0.05).

To investigate the effect of distribution sites on the rhizosphere microbial community of *C. chinensis* var. *brevisepala*, alpha diversity, which reflects microbial community diversity and richness, was analyzed. Significant differences in alpha diversity were observed across sites. For the bacterial community, the ACE index at site PA was significantly higher than at GHZ and HH, while no significant inter-site differences were observed in Shannon index. For the fungal community, the ACE indices at sites LTS, GHZ, and HH were significantly higher than at PA, and the Shannon index at LTS was significantly higher than at PA (Figures 2A–D).

Principal Coordinates Analysis (PCoA) based on Bray-Curtis distances showed that microbial communities within the same site were well-clustered, with significant separation observed between sites (bacteria: *R*^2^ = 0.721, *p* = 0.001; fungi: *R*^2^ = 0.807, *p* = 0.001) (Figures 2E,F). Notably, GHZ and HH showed minimal inter-group differences and were separated from PA and LTS on opposite sides of the PC1 axis. Additionally, heatmap analysis of genus-level relative abundances of bacterial and fungal communities across sites further confirmed these inter-site microbial differences (Supplementary Figure S1).

Linear discriminant analysis effect size (LEfSe) (LDA > 4) was further performed to identify location-specific microbial taxa across different distribution sites. For the bacterial community, LEfSe results showed that 2, 5, 3, and 5 bacterial genera were significantly enriched at PA, LTS, GHZ, and HH, respectively (Figure 2G). *Bacillus* and *Pseudomonas* were the dominant enriched genera at PA; *Flavobacterium*, mle1-7, norank_c_bacteriap25, norank_f_Methyloligellaceae, and norank_f_SC-I-84 at LTS; *Acidothermus*, *Acidibacter*, and *Bradyrhizobium* at GHZ; and *Burkholderia-Caballeronia-Paraburkholderia*, *Roseiarcus*, norank_f_Xanthobacteraceae, norank_o_Acidobacteriales, and norank_o_Subgroup_2 at HH. For the fungal community, a total of 15 genera were identified as biomarkers, with 2, 5, 6, and 2 biomarkers specific at PA, LTS, GHZ, and HH, respectively (Figure 2H). *Mycoarthris* and *Paraboeremia* at PA; *Calycina*, *Coniothyrium*, Fungi_gen_Incertae_sedis, *Metarhizium*, and *Plectosphaerell* at LTS; *Deconica*, GS11_gen_Incertae_sedis, *Mortierella*, *Penicillium*, *Saitozyma*, and *Solicoccozyma* at GHZ; and *Keithomyces* and *Podila* at HH.

### 3.3 Soil chemical properties and their correlation with rhizosphere microbial community structure

Table 1 lists the chemical properties of soils among the four sites. Soils at all sites were acidic, and the pH values at PA and HH were significantly higher than those at GHZ and LTS. PA exhibited the highest contents of total phosphorus (TP, 1.05 ± 0.06 g/kg), available phosphorus (AP, 173.31 ± 6.98 mg/kg), and available potassium (AK, 363.70 ± 5.20 mg/kg); in contrast, LTS had the lowest TP (0.52 ± 0.11 g/kg) and AP (10.08 ± 1.54 mg/kg). Total nitrogen (TN) contents at PA (2.97 ± 0.07 g/kg) and LTS (3.22 ± 1.28 g/kg) had significantly lower TN than GHZ (8.19 ± 2.31 g/kg) and HH (7.66 ± 1.77 g/kg). No significant difference in soil organic matter (SOM) content was observed among the four sites. Additionally, soil properties at GHZ and HH were slightly different, with both sites characterized by strongly acidic soils and notably higher TN contents compared to PA and LTS.

**Table 1:** Contents of some major soil nutrients and soil pH in different distribution sites *C. chinensis* var. *brevisepala.*

Soil type	PA	LTS	GHZ	HH	*F*-value	*p*-value
pH	5.39 ± 0.07a	5.24 ± 0.28a	3.42 ± 0.03b	3.72 ± 0.19b	103.49	<0.001
SOM (%)	4.22 ± 0.12a	3.57 ± 1.36a	5.48 ± 0.72a	5.73 ± 0.84a	4.10	0.049
TON (g/kg)	2.97 ± 0.07b	3.22 ± 1.28b	8.19 ± 2.31a	7.66 ± 1.77a	9.29	0.006
TOP (g/kg)	1.05 ± 0.06a	0.52 ± 0.11b	0.58 ± 0.07b	0.65 ± 0.11b	22.22	<0.001
TOK (g/kg)	10.18 ± 1.00b	14.15 ± 3.71ab	22.06 ± 6.00a	13.50 ± 1.36ab	5.79	0.021
AN (mg/kg)	99.17 ± 2.67c	117.25 ± 22.89bc	210.00 ± 59.16ab	262.50 ± 49.62a	11.05	0.003
AP (mg/kg)	173.31 ± 6.98a	10.08 ± 1.54c	24.90 ± 5.71b	22.36 ± 0.61b	854.94	<0.001
AK (mg/kg)	363.70 ± 5.20a	215.35 ± 77.36b	256.48 ± 44.44ab	167.22 ± 48.53b	8.13	0.008

Mean value (*n* = 3) ± STDEV followed by different letters indicate a significant difference (*p* < 0.05, Trukey HSD test).

**Figure 3:**
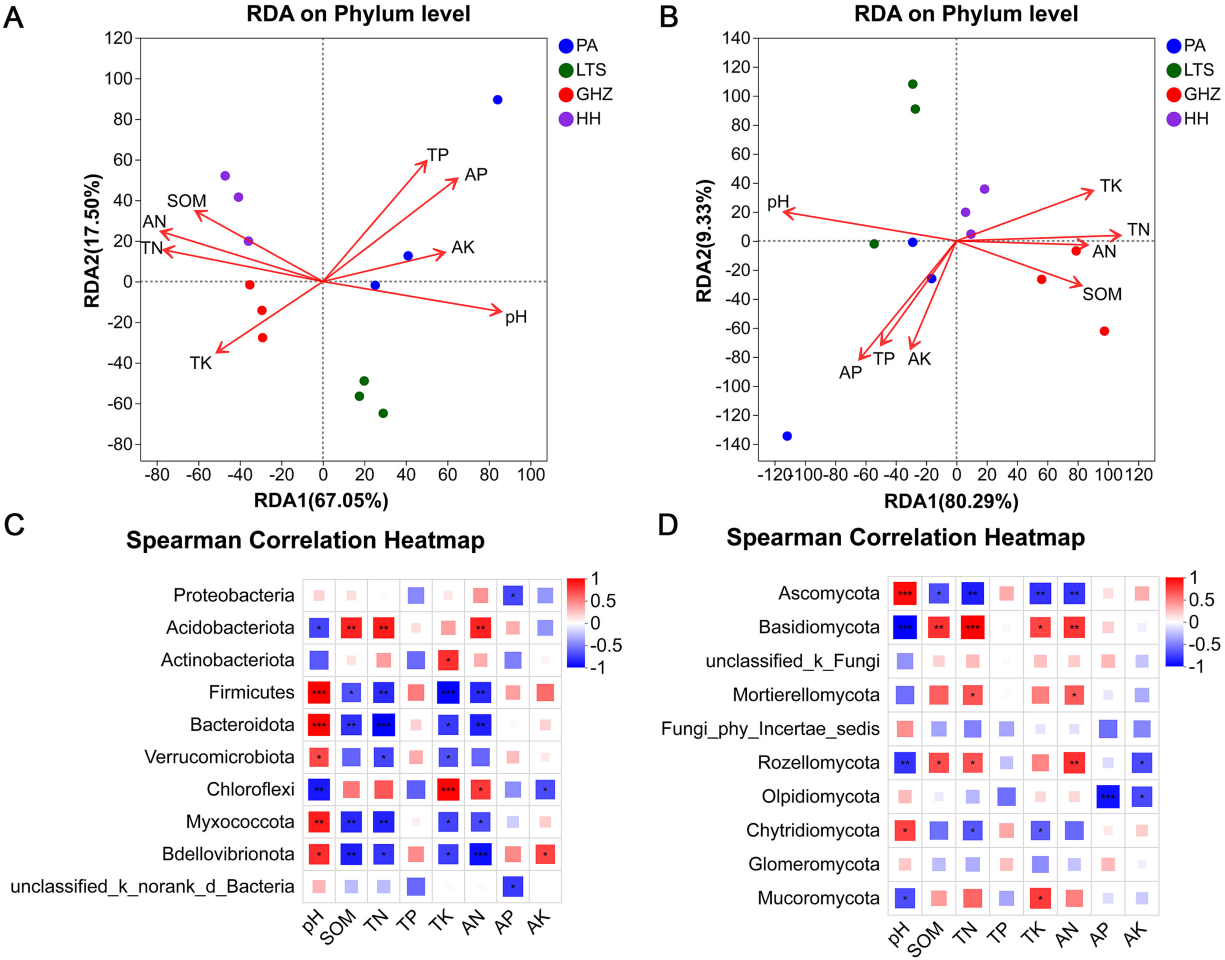
Correlation between soil properties and rhizosphere microbial community structure of *C. chinensis* var. *brevisepala* across multiple sites. **(A,B)** RDA ordination plots showing the relationship of the bacterial **(A)** and fungal **(B)** community structure with soil physicochemical properties. The position and length of the arrows indicate the direction and strength of the influence of soil variables on microbial communities, respectively. **(C,D)** Spearman correlation heatmap of bacteria **(C)** and fungi **(D)** at the phylum level with soil physicochemical properties (*p ≦ 0.05; **p ≦ 0.01; ***p ≦ 0.001. The same below).

Correlation analyses were conducted between the relative abundances of bacteria/fungi and soil chemical properties. Redundancy analysis (RDA) at the phylum level indicated that besides SOM and TP (*p* < 0.05), soil pH, TN, AN, and AP were the dominant factors influencing bacterial community structure (*p* < 0.01; Figure 3A; Supplementary Table S1). Fungal community structure was affected by soil factors including pH and TN (*p* < 0.01; Figure 3B; Supplementary Table S1). Collectively, soil properties drove variations in the rhizosphere bacterial and fungal communities of *C. chinensis* var. *brevisepala*, with fungal communities being more sensitive to soil conditions (the explanatory power of RDA1 in Figure 3B exceeded 80%, higher than the 67% observed for bacteria in Figure 3A).

Spearman correlation heatmaps were used to clarify correlations between dominant microbial phyla and soil factors (Figures 3C,D). For bacteria, most dominant phyla showed significant correlations with soil pH: Firmicutes, Bacteroidota, Verrucomicrobiota, Myxococcota, and Bdellovibrionota were posotively correlated, while Chloroflexi and Acidobacteriota were negatively correlated. Additionally, Firmicutes, Bacteroidota, Myxococcota, and Bdellovibrionota exhibited significant negative correlations with SOM, TN, TK, and AN; in contrast, Acidobacteriota showed strong positive correlations with SOM, TN, and AN, and Chloroflexi displayed positive correlations with TK and AN. For fungi, Ascomycota was strongly positively correlated with soil pH and significant negatively correlated with SOM, TN, TK, and AN. In contrast, Basidiomycota exhibited the opposite trend: strong negative correlation with pH and significantly positive correlations with SOM, TN, TK, and AN. Other fungal phyla, including Mortierellomycota, Rozellomycota, Chytridiomycota, and Mucoromycota also exhibited certain correlations with soil chemical properties.

### 3.4 Functional prediction of rhizosphere microbial community

**Figure 4:**
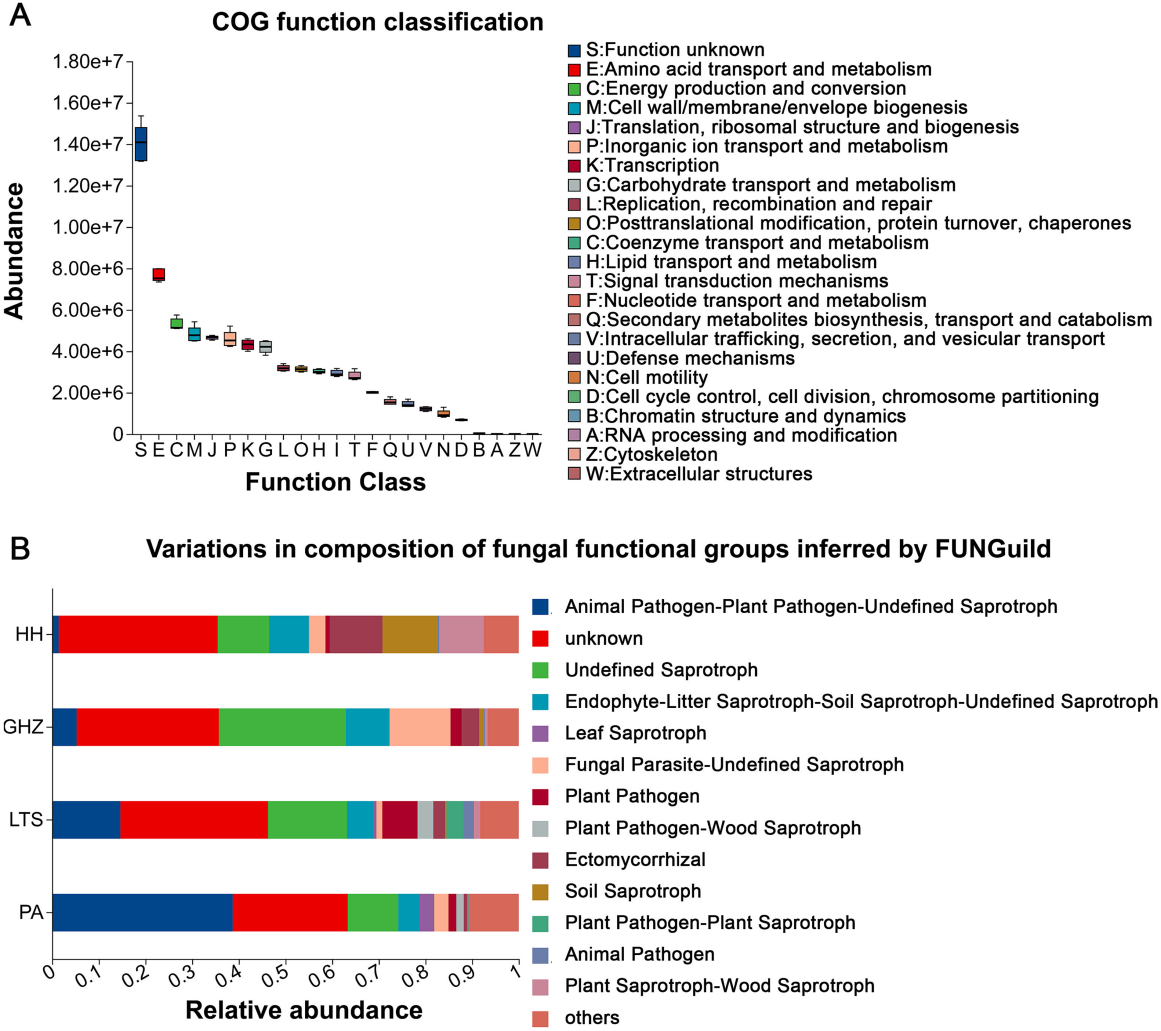
Functional profiles of rhizosphere microbial communities of *C. chinensis* var. *brevisepala* across different locations: **(A)** Functional features of the bacterial community and **(B)** functional features of the fungal community. The COG classification is represented by the abscissa, and the ordinate indicates the abundance of the function.

To clarify the microecological functions of rhizosphere microorganisms in *C. chinensis* var. *brevisepala* rhizosphere soil, PICRUSt2 was used to predict the functional profiles of bacterial communities (Figure 4A). Among the identified functional categories, metabolism-related functions accounted for the highest proportion, including “Amino acid transport and metabolism,” “Energy production and conversion,” “Translation, ribosomal structure and biogenesis,” “Inorganic ion transport and metabolism,” “Transcription, and Carbohydrate transport and metabolism.” Although the types of enriched bacterial functional categories were consistent across all sites, the analysis did not account for site-specific differences in bacterial relative abundance. Notably, the relative frequency of major functional traits was highest in the GHZ microbial community and lowest in LTS.

Subsequently, the FUNGuild tool was employed to predict fungal community functions (Figure 4B). “Animal Pathogen-Plant Pathogen-Undefined Saprotroph” was the most abundant functional category, followed by “Undefined Saprotroph and Endophyte-Litter Saprotroph-Soil Saprotroph-Undefined Saprotroph.” The relative abundance of “Animal Pathogen-Plant Pathogen-Undefined Saprotroph” at PA was significantly higher than that at the other three sites, whereas PA exhibited the lowest abundance of “Undefined Saprotroph” and “Endophyte-Litter Saprotroph-Soil Saprotroph-Undefined Saprotroph.” Additionally, the relative abundances of “Fungal Parasite-Undefined Saprotroph” at GHZ, and “Ectomycorrhizal” and “Soil Saprotroph” at HH were significantly higher than those at other sites. Additionally, among the 6,873 fungal ASVs, 386 were assigned with high-confidence predictions. These ASVs were classified into five trophic modes: symbiotroph (65.03%), saprotroph (31.09%), pathotroph (2.33%), pathotroph-saprotroph (1.30%), and saprotroph-symbiotroph (0.26%) (Supplementary Table S2).

### 3.5 Composition and diversity analysis of endophytes in leaves, rhizomes, and fibrous roots of *Coptis chinensis* var. *brevisepala*

**Figure 5:**
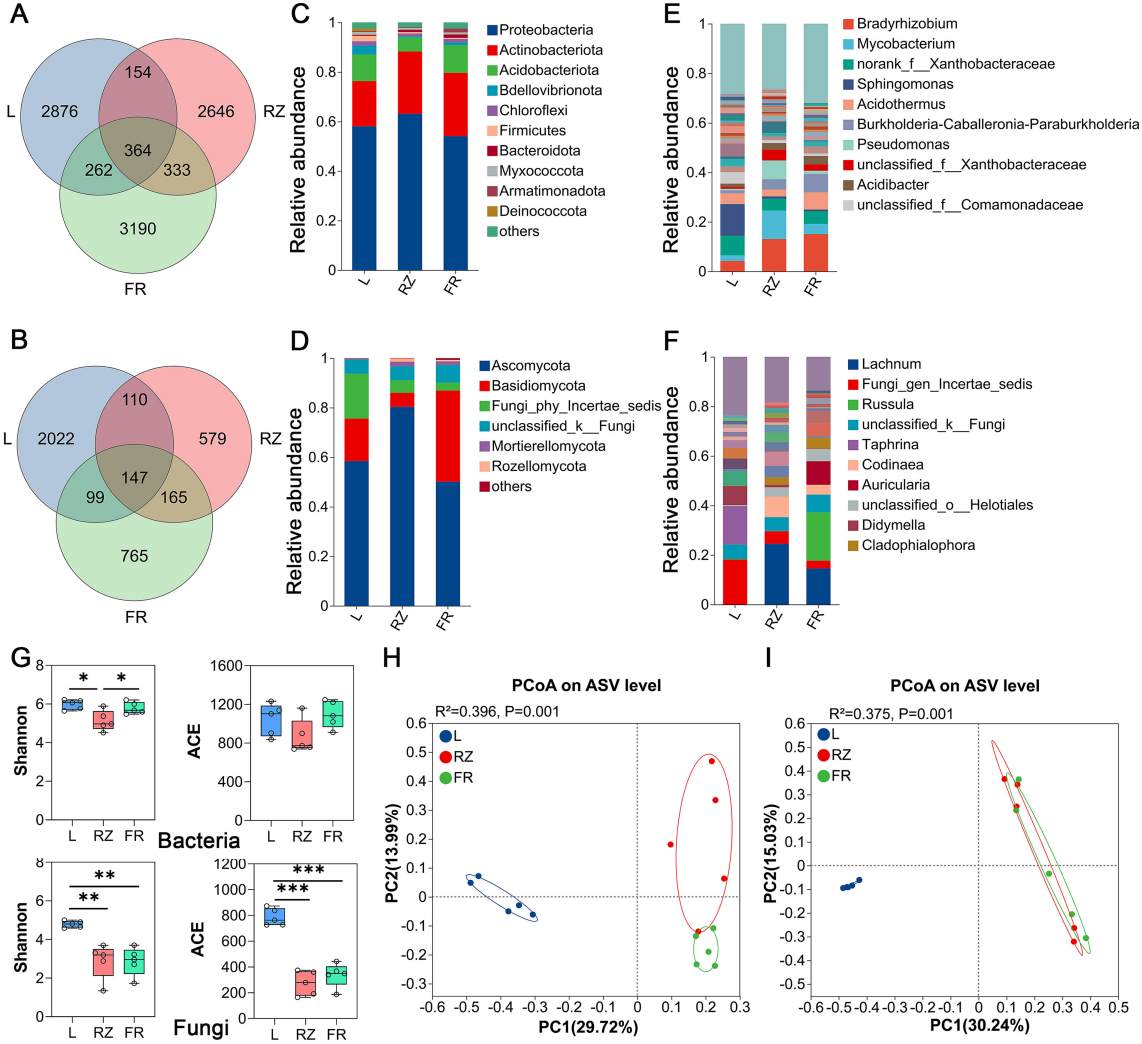
Microbial composition and diversity analysis of the leaves, rhizomes, and fibrous roots of *C. chinensis* var. *brevisepala*. **(A,B)** The endophytic bacteria **(A)** and fungi **(B)** in the leaves, rhizomes, and fibouse roots of *C. chinensis* var. *brevisepala* unique and common ASV; **(C,D)** The relative abundance of endophytic bacteria **(C)** and fungi **(D)** at the phylum level; **(E,F)** The relative abundance of endophytic bacteria **(E)** and fungi **(F)** at the genus level; **(G)** Alpha diversity index of endophyte of leaves, rhizomes, and fibouse roots; **(H,I)** PCoA analysis of endophytic bacteria **(H)** and fungi **(I)** (L, Leaf; RZ, rhizomes; FR, fibrous roots; *p ≤ 0.05; **p ≤ 0.01; ***p ≤ 0.001).

Based on the ASV level, a venn diagram were used to show the ASV number of unique endophytes and common endophytes in each tissue of *C. chinensis* var. *brevisepala* (Figures 5A,B). For further analysis of species composition, the top taxa at the phylum and genus levels were classified (Figures 5C–F). A total of 29 phyla and 596 genera were identified for endophytic bacteria, while 12 phyla and 653 genera were detected for endophytic fungi. Excluding unclassified and other taxa, the top 3 dominant endophytic bacterial phyla across all tissues were Proteobacteria (58.36%), Actinobacteriota (23.02%), and Acidobacteriota (9.23%), with the remaining phyla accounting for <4%. For endophytic fungi, the top 3 dominant phyla were Ascomycota (62.95%), Basidiomycota (19.86%), and Fungi_incertae_sedis (8.79%), with other phyla each accounting for <2%. At the genus level, the top 4 endophytic bacteria in leaves were *Sphingomonas* (12.80%), norank_f_Xanthobacteraceae (7.90%), unclassified_f_Comamonadaceae (4.60%), and *Acidothermus* (4.36%); in rhizomes, they were *Bradyrhizobium* (13.10%), *Mycobacterium* (11.51%), *Pseudomonas* (7.55%), and norank_f_Xanthobacteraceae (4.78%); in fibrous roots, they were *Bradyrhizobium* (15.11%), *Burkholderia-Caballeronia-Paraburkholderia* (7.43%), *Acidothermus* (7.00%), and norank_f_Xanthobacteraceae (5.07%). For endophytic fungi, Fungi_gen_Incertae_sedis (18.15%), *Taphrina* (15.53%), and *Didymella* (7.67%) were the most abundant in leaves; *Lachnum* was dominant in rhizomes (24.49%), followed by fibrous roots (14.49%), but barely detectable in leaves (0.01%); *Russula* (19.47%) and *Auricularia* (9.50%) were most abundant in fibrous roots yet nearly undetectable in rhizomes and leaves.

Figure 5G showed that the Shannon index of endophytic bacterial communities in rhizomes was significantly lower than that in leaves and fibrous roots, indicating higher endophytic bacterial diversity in leaves and fibrous roots. Regarding endophytic fungi, both the Shannon and ACE indices in leaves were significantly higher than those in rhizomes and fibrous roots, suggesting greater fungal diversity and richness in leaves. No significant differences in bacterial ACE indices were observed among tissues, implying similar bacterial richness across different plant tissues.

PCoA revealed significant differences in the overall structure of endophytic communities among leaves, rhizomes, and fibrous roots (Figures 5H,I). Further pairwise comparisons indicated significant structural differences in endophytic bacterial and fungal communities between all tissue pairs, except for no significant difference in endophytic fungal communities between rhizomes and fibrous roots (Supplementary Figure S2).

### 3.6 Relationships between endophytes and rhizosphere microorganisms, and functional prediction of endophytes

By screening the common microorganisms of the rhizosphere core microbiota and endophytic core microbiota of *C. chinensis* var. *brevisepala*, 153 genera of common bacteria and 75 common fungi were identified and defined as common core bacteria and common core fungi (Supplementary Table S3), suggesting a potential soil origin for endogenous common core dominant bacteria and fungi.

**Figure 6:**
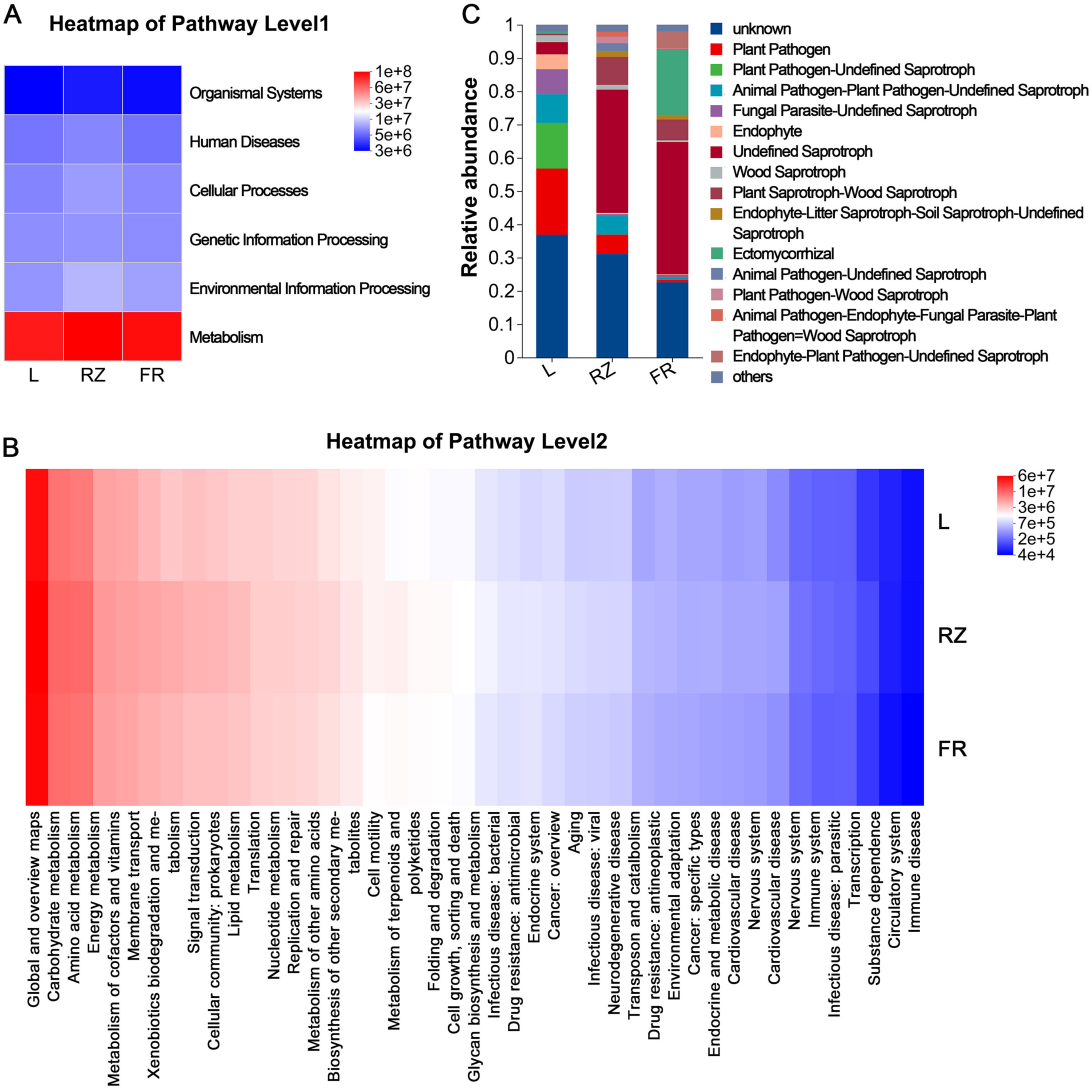
Functional profiles of endophytic communities in *C. chinensis* var. *brevisepala*. **(A)** Abundance of KEGG first-level functional genes of endophytic bacteria. **(B)** Abundance of KEGG second-level functional genes of endophytic bacteria. **(C)** FUNGuild analysis of endophytic fungi.

Functional prediction of endophytic bacterial communities was performed using PICRUSt2. Based on the abundance distribution of primary functional genes in leaves, rhizomes, and fibrous roots, metabolic-related genes accounted for the highest abundance among the 6 known biological metabolic pathways (Figure 6A), indicating metabolism as the primary function. For secondary functional genes, endophytic bacteria exhibited diverse metabolic functions, including basic substance metabolism (Carbohydrate metabolism, Amino acid metabolism), specialized metabolite synthesis and metabolism (Biosynthesis of other secondary metabolites, Metabolism of terpenoids and polyketides), and metabolism-related processes (Folding and degradation, Membrane transport) (Figure 6B). Additionally, endophytic bacteria in various tissues displayed functions such as microbial resistance, suggesting potential roles in defending against certain pathogenic microorganisms. FUNGuild-based functional prediction of endophytic fungal communities in leaves, rhizomes, and fibrous roots indicated that saprotrophic functions (e.g., plant pathogens) were most abundant (Figure 6C).

## 4 Discussion

### 4.1 Diversity and composition of the rhizosphere microbial communities

The present study examined rhizosphere soils of *C. chinensis* var. *brevisepala* from four field sites and identified 177 core bacterial genera spanning 20 phyla, with Proteobacteria, Acidobacteriota, Actinobacteriota, Firmicutes, and Bacteroidota as the dominant phyla (Figure 1C). These core bacteria across the four sites are not easily disturbed by environmental factors, and the majority are beneficial bacteria, suggesting that they may be resident bacteria in *C. chinensis* var. *brevisepala* rhizosphere soil. Notably, Acidobacteriota is a highly prevalent bacterial phylum found in soils, and its members are reported to participate in nutrient cycling and organic matter decomposition (Gonçalves et al., 2024; McReynolds et al., 2025). The core bacterial genera identified herein mainly include norank_f_Xanthobacteraceae, *Bradyrhizobium*, *Bacillus*, norank_o_Subgroup_2, and norank_o_Acidobacteriales (Figure 1E). These taxa are resilient to environmental fluctuations and play crucial roles in maintaining the biological functions of the host plant. Most of these bacteria exert beneficial effects on *C. chinensis* var. *brevisepala* by promoting growth and stabilizing the rhizosphere microecosystem via multiple mechanisms. For instance, *Bradyrhizobium*, a typical nitrogen fixer, can convert atmospheric nitrogen into plant-available ammonium, thereby increasing rhizosphere nitrogen content to sustain plant nutrition (Sarao et al., 2025). *Bacillus* can directly facilitate plant growth by synthesizing hormones such as indole-3-acetic acid (IAA) and inhibit rhizosphere pathogens via antimicrobial peptides, ultimately reducing disease risk (Karacic et al., 2024).

Regarding fungi, 146 core genera across 11 phyla were detected, with Ascomycota and Basidiomycota as the dominant phyla (Figure 1D). This result is consistent with previous findings on the rhizosphere fungal community of *Coptis chinensis* (Wu et al., 2023). *C. chinensis* var. *brevisepala* naturally grows in understory habitats with abundant leaf litter (Chen and Liu, 2012), where soil microbes regulate the decomposition of plant residues. Previous studies have confirmed that Ascomycota and Basidiomycota play key roles in decomposition of organic matter, particularly lignin and cellulose (Jia et al., 2025). This suggests that these dominant fungal phyla may also be involved in soil carbon cycling of *C. chinensis* var. *brevisepala* habitats, driving organic matter decomposition and carbon supply.

These core taxa were stably distributed across all sampling sites and constituted the conserved core framework of microbiomes in distinct habitats. Spatial variation in their abundance further shaped site-specific biomarkers. Biomarkers identified by LEfSe analysis clearly resolved community differentiation of microbiomes across PA, LTS, GHZ, and HH. Notably, several core taxa displayed both broad ubiquity and strong habitat specificity, acting as key hubs connecting the conserved core microbiome structure to spatial community differentiation (Figures 2G,H). For example, *Bacillus* and *Paraboeremia* served as both core genera and site-specific biomarker at the PA site.

Currently, *C. chinensis* var. *brevisepala* is an endangered species and urgently requires conservation intervention. By analyzing the rhizosphere microbial communities of *C. chinensis* var. *brevisepala* across four distinct sites, this study identified beneficial microbial taxa enriched by the host plant, thereby providing a microbe-oriented strategy to guide future conservation efforts for this species.

### 4.2 The relationship between rhizosphere microbial communities and soil factors

Ecological drivers vary in their ability to shape rhizosphere microbial community composition. Among these, soil properties are well-recognized as the key determinants of soil microbiome assembly (Eichorst and Kuske, 2012). Considerable effort has been dedicated that the diversity and composition of rhizosphere microbial communities are modulated by a series of soil factors, including pH, fertility, and soil moisture (Rousk et al., 2010; Wan et al., 2020; Xiong et al., 2024). In present study, significant regional variations were observed in soil chemical properties, particularly pH, TP, AP (Table 1). The RDA results clearly indicated that soil pH and TN were the most critical factors driving variation in both fungal and bacterial community structures (Figures 3A,B; Supplementary Table S1). The pH of all samples in this study ranged from 3.42 to 5.39, corresponding to strongly acidic conditions. A negative correlation was observed between the relative abundance of Acidobacteriota and soil pH, which is consistent with previous findings (Lauber et al., 2009). Conversely, Firmicutes, Bacteroidota, Verrucomicrobiota, Myxococcota, and Bdellovibrionota exhibited positive correlations with pH, suggesting that these phyla favor acidic environments for survival. Additionally, the relative abundance of Acidobacteriota was positively correlated with SOM, TN, and AN (*p* < 0.01), suggesting that this phylum thrives in nutrient-rich habitats and could serve as an indicator of soil fertility (Figure 3C). Furthermore, Ascomycota and Basidiomycota exhibited significant associations with soil factors: Ascomycota correlated negatively with SOM, TN, TK, and AN, while Basidiomycota displayed a significant positive correlation with these nutrient contents (Figure 3D). This reflects divergent ecological adaptabilities to soil nutrient conditions between the two dominant fungal phyla: Ascomycota tends to be enriched in relatively nutrient-poor soils, whereas Basidiomycota thrived in soils with high organic matter and nutrient reserves. Given the saprophytic metabolism of most Basidiomycota taxa, we infer this phylum plays a pivotal role in decomposing complex organic matter (e.g., lignin and cellulose) and regulating nutrient cycling processes. In summary, this study confirms soil factors act as key environmental drivers of structural and functional differentiation in the core rhizosphere microbial community in *C. chinensis* var. *brevisepala*. The differential nutrient responses and functional complementarity among core taxa collectively sustain the stability and functionality of the rhizosphere microecosystem. These findings provide a theoretical basis for optimizing *C. chinensis* var. *brevisepala*’s growing environment through soil nutrient management or functional microbe inoculation.

### 4.3 Composition and function of the endophytes

High-throughput sequencing technology was employed to characterize the community structure and diversity of endophytic bacteria and fungi in *C. chinensis* var. *brevisepala*. Previous studies have demonstrated that endophytes exhibit significant tissue specificity, with the dominant genera and diversity levels varying across different plant tissues (Arora et al., 2019; Kumar and Prasher, 2022; Ming et al., 2022; Xu Q. et al., 2024). Consistent with this, the highest endophytic diversity—particularly for fungi—was detected in leaves in the present study (Figure 5G). Furthermore, PCoA confirmed obvious tissue specificity of endophytes in *C. chinensis* var. *brevisepala* (Figures 5H,I). In addition, the community structure of endophytes in leaves was significantly divergent from those of rhizomes or fibrous roots (Figures 5C–F). Such prevalent inter-tissue differences in endophyte diversity across plants may arise from tissue-specific microenvironments and unique community traits shaped by long-term environmental adaptation.

At the phylum level, Proteobacteria (58.36%), Actinobacteriota (23.02%), and Acidobacteriota (9.23%) dominated the endophytic bacteria in leaves, rhizomes, and fibrous roots of *C. chinensis* var. *brevisepala* (Figure 5C). At the genus level, *Bradyrhizobium* maintained a high abundance across all three tissues (Figure 5E), which is consistent with previous findings that this genus is highly abundant in the fibrous roots of *C. chinensis* var. *brevisepala* across different growth years (Dai et al., 2026). Notably, *Bradyrhizobium* also exhibited a high relative abundance in the rhizosphere soil of *C. chinensis* var. *brevisepala* (Figure 1E), highlighting the crucial role of this beneficial bacterium in the growth of *C. chinensis* var. *brevisepala*. For endophytic fungi, Ascomycota (62.95%) and Basidiomycota (19.86%) were the dominant phyla (Figure 5D), consistent with reports on endophytic fungal communities in most plants (Du et al., 2020). Meanwhile, several endophytic fungal genera showed distinct tissue specificity: *Russula* was significantly enriched in fibrous roots but nearly undetectable in leaves and rhizomes (Figure 5F). Multiple studies have indicated that *Russula* acts as an ectomycorrhizal fungus, forming symbiotic relationships with plants and contributing to nutrient cycling in forest ecosystems (Yu et al., 2021; Yang et al., 2025). Its high relative abundances in fibrous roots suggests that *Russula* may regulate the nutrient absorption process of the plant’s root system through synergistic effects, thereby promoting plant growth. *Lachnum* exhibited the highest relative abundance in rhizomes (24.49%), followed by fibrous roots (14.49%), and was extremely rare in leaves (Figure 5F). *Lachnum* holds great research and application value, as it can produce various bioactive substances such as polysaccharides and polyphenols. Notably, rhizomes are the medicinal part of *C. chinensis* var. *brevisepala,* rich in characteristic alkaloid components like berberine. Thus, the high abundance of *Lachnum* in rhizomes may contribute to the high accumulation of alkaloid-type medicinal components in this tissue. Based on functional prediction and tissue distribution, *Bradyrhizobium*, *Russula* and *Lachnum* are proposed as priority genera for further experimental verification, which is crucial for clarifying their real functions in promoting plant growth and medicinal ingredient accumulation, and provides a key basis for developing beneficial endophyte inoculants.

## 5 Conclusion

This study systematically analyzed the microbial diversity of rhizosphere soils and plant tissues of *C. chinensis* var. *brevisepala* collected from four geographic sites. Our findings identify both shared and site-specific beneficial microbial taxa enriched in the rhizosphere across the four study sites. Soil chemical properties, particularly the soil pH and total nitrogen, exert a significant impact on the rhizosphere microbiomes, with stronger correlations observed between these soil factors and bacterial communities than with fungal communities. Endophytes of *C. chinensis* var. *brevisepala* exhibit significant tissue specificity, with leaf endophytes showing the highest diversity. A total of 199 core bacterial genera and 105 core fungal genera are shared across different tissues of this plant, and these shared core endophytes display a high degree of overlap with the core rhizosphere soil microbial taxa. Functional prediction indicates that the endophytes of *C. chinensis* var. *brevisepala* possess potential functions in regulating plant growth and secondary metabolism. Collectively, this study identifies highly enriched core rhizosphere microorganisms and growth-linked tissue-specific endophytes, elucidating the species’ adaptive mechanisms and establishing a robust theoretical basis for its ecological restoration. Furthermore, the specific functions and intrinsic regulatory mechanisms by which *Bradyrhizobium*, *Russula*, and *Lachnum* mediate the growth and environmental adaptation of *C. chinensis* var. *brevisepala* merit further in-depth exploration.

## Data Availability

The data of this study are publicly available at https://ngdc.cncb.ac.cn (Genome Sequence Archive, accession no. PRJCA058903). Further inquiries can be directed to the corresponding author.

## Footnotes

1 https://github.com/OpenGene/fastp, v0.19.6.

2 https://ccb.jhu.edu/software/FLASH/index.shtml, v1.2.11.

3
https://www.majorbio.com/tools

4
http://www.mothur.org/wiki/Calculators

5
http://huttenhower.sph.harvard.edu/LEfSe
