# Supplementary material for: The structure of rhizosphere microbial and endophytic communities of Coptis chinensis var. brevisepala: variations across different ecological niches
Source: Front Microbiol. 2026 Mar 18;17:1785609. doi: 10.3389/fmicb.2026.1785609 (PMC13038877; doi:10.3389/fmicb.2026.1785609)
Supplement: Supplementary file 1 [file Supplementary_file_1.DOCX]

Supplementary Material


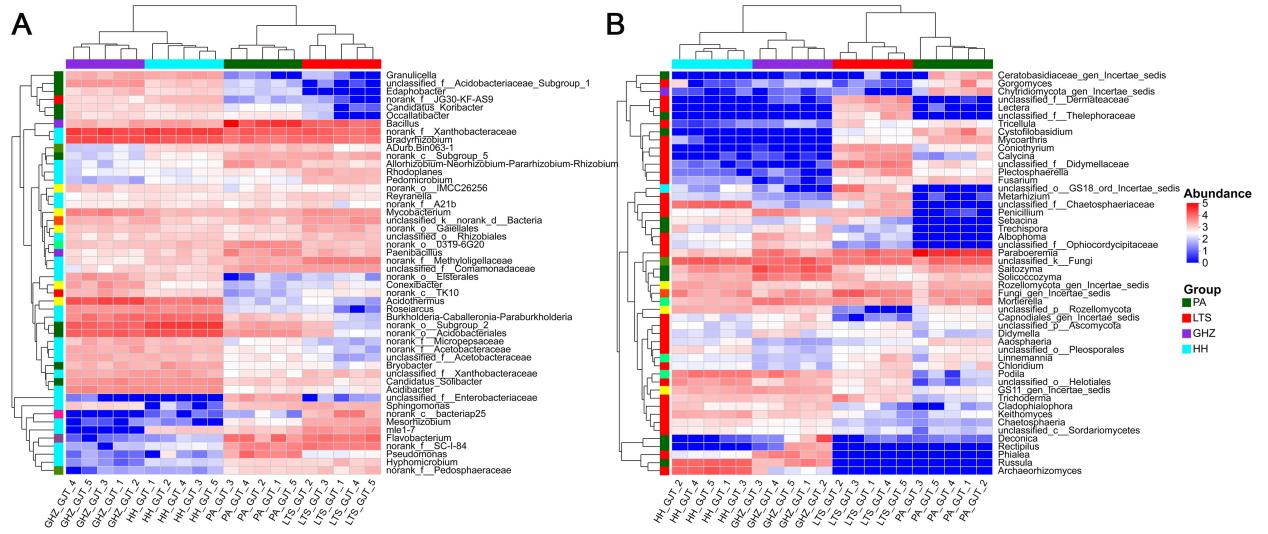


**Supplementary Figure 1.** Heatmap of relative abundance of bacterial and fungal genera of *C. chinensis* var. *brevisepala* in different distribution sites.

**Supplementary Table 1**. Relationships of bacterial and fungal community compositions with soil factors identified based on RDA analysis.

|  | **pH** | **SOM** | **TN** | **TP** | **TK** | **AN** | **AP** | **AK** |
| --- | --- | --- | --- | --- | --- | --- | --- | --- |
| **Bacterial** |  |  |  |  |  |  |  |  |
| RDA1 | 0.987 | -0.905 | -0.983 | 0.722 | -0.883 | -0.964 | 0.852 | 0.986 |
| RDA2 | -0.160 | 0.426 | 0.183 | 0.692 | -0.470 | 0.266 | 0.524 | 0.170 |
| r^2^ | 0.817** | 0.515* | 0.658** | 0.553* | 0.371 | 0.715** | 0.654** | 0.378 |
| *P* values | 0.004 | 0.039 | 0.005 | 0.025 | 0.120 | 0.004 | 0.006 | 0.119 |
| **Fungal** |  |  |  |  |  |  |  |  |
| RDA1 | -0.980 | 0.940 | 0.978 | -0.626 | 0.999 | 0.996 | -0.677 | -0.393 |
| RDA2 | 0.200 | -0.341 | -0.208 | -0.780 | 0.0400 | -0.087 | -0.736 | -0.920 |
| r^2^ | 0.730** | 0.419 | 0.660** | 0.336 | 0.432 | 0.3990 | 0.482 | 0.276 |
| *P* values | 0.001 | 0.100 | 0.007 | 0.170 | 0.103 | 0.098 | 0.053 | 0.235 |

Note: SOM, soil organic matter; TN, total nitrogen content; TP, total phosphorus content; TK, total potassium content; AN, available N content; AP, available P content; AK, available K content. * *P* < 0.05, ** *P* < 0.01, ANOVA.


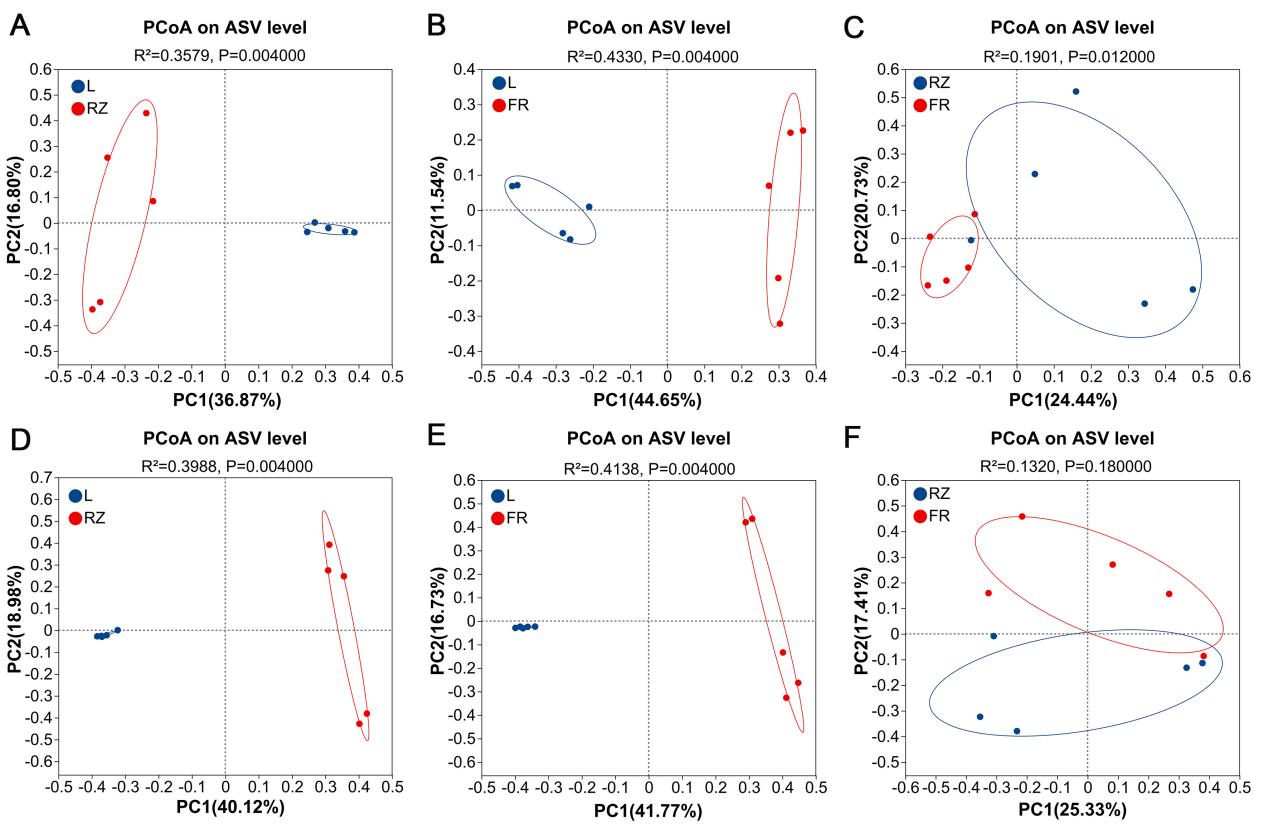


**Supplementary Figure 2.**Pair-to-pair comparative analysis of endophytic bacteria and fungi PCoA in leaves, rhizomes, and fibrous roots of *C. chinensis* var. *brevisepala.*
